# Mapping the landscape of rural cancer research: a global bibliometric analysis

**DOI:** 10.1007/s10552-025-02086-0

**Published:** 2025-12-27

**Authors:** David Nelson, Tanja K. Kleinhappel, Natalia Calanzani, Samuel Cooke, Ben Pickwell-Smith, Katie Spencer, Ros Kane, Shahana A. Naqvi, Peter Selby, Mark Lawler, Peter Murchie

**Affiliations:** 1 Lincoln Institute for Rural and Coastal Health, College of Health and Science, Lincoln Medical School, University of Lincoln, Brayford Pool, Lincoln, LN6 7TS UK; 2 Macmillan Cancer Support, London, UK; 3 Academic Primary Care Research Group, Institute of Applied Health Science, University of Aberdeen, Aberdeen, UK; 4 Leeds Cancer Centre, Leeds Teaching Hospitals NHS Trust, Leeds, UK; 5 Faculty of Medicine and Health, School of Medicine, University of Leeds, Leeds, UK; 6 Leeds Institute of Health Sciences, Faculty of Medicine and Health, University of Leeds, Leeds, UK; 7 School of Health and Care Sciences, College of Health and Science, University of Lincoln, Lincoln, UK; 8 Lincoln Medical School, Universities of Nottingham and Lincoln, Lincoln, UK; 9 Johnston Cancer Research Centre, Faculty of Medicine, Health and Life Sciences, Queens University Belfast, Belfast, UK; 10 European Cancer Organisation, Brussels, Belgium

**Keywords:** Cancer research, Rural health, Bibliometric analysis, Research activity, Global, Inequalities

## Abstract

**Purpose:**

Rural communities frequently experience inequalities in cancer care compared to urban counterparts. Despite growing academic interest**,** there has been no global bibliometric analysis of rural cancer publications. Increasingly, global policies focus on place-based health inequalities; it is critical to understand the current state and emerging trends of rural cancer research. This analysis focuses on publication trends, including authors, citations, geography, collaboration (extent and patterns) and target journals.

**Methods:**

Web of Science and Scopus were searched from inception to 25th February 2025. Bibliometric methodology examined citation counts, authorship and publication sources. Results were converted into bibliographic data frames using the *bibliometrix* R package. All analysis and visual illustrations were in R 4.4.2.

**Results:**

Fifteen thousand seven hundred and twenty two documents were analysed (mean age, 10.6 years; average, 25.5 citations per document (2.2 per year)). Annual publication growth was 4.6%, with a marked increase in rural cancer research outputs since 2006. Research output was concentrated in a small number of high-income countries and institutions, but citation analysis showed that some smaller countries produced high-impact work. Rural cancer research activity is shaped by national, regional and geopolitical collaborations. Thematic gaps were identified in early diagnosis. Cancer-specific journals have most outputs, with rural health and public health journals also contributing to the dissemination of rural cancer research.

**Conclusion:**

Rural cancer research is expanding but is geographically uneven. There is a need for increased investment in underrepresented regions and broader subject-specific coverage that is guided by intersectional and place-based approaches.

**Supplementary Information:**

The online version contains supplementary material available at 10.1007/s10552-025-02086-0.

## Introduction

Global health policy agendas are increasingly focused on addressing cancer inequalities among underserved communities, such as people who live in rural/remote areas. It is widely acknowledged that rural communities frequently experience inequalities throughout the cancer journey, compared to their urban counterparts [1–7]. People with cancer from rural areas have lower screening uptake [8, 9], more advanced disease at diagnosis [10–12], and poorer long-term survival [13], when compared to those from urban areas. Globally, 43% of people reside in rural areas; across the European continent it averages 27%, with Canada at 18% and the United States of America (USA) at 17% [14]. Despite an emerging body of evidence in the United Kingdom (UK) [15–19] and Europe [20, 21], the academic study of rurality and cancer has been dominated by scholars from Asia, Australia, and North America [5, 13].

There is no universal definition or measure of rurality; conceptually, ‘rural’ can mean different things to different communities worldwide [22]. What constitutes rurality in large land masses such as Australia, Canada, or the USA could differ considerably in terms of scale and remoteness, compared to relatively smaller countries across the European continent. However, there is some consensus that rural communities approach their health differently and hold unique views on defining and managing their health compared to non-rural communities [23]. As such, if evidence-based national and global policy is to address these inequalities, the increased policy focus must be mirrored by commensurate growth in scientific activity in the rural oncology domain. Therefore, it is critical to understand the current state and emerging trends of rural cancer research activity at a global level.

Bibliometrics involves a range of methods for the analysis of scientific publications [24]. It provides a comprehensive overview and insight into a discipline, field or subject [25]. There are a number of bibliometric analyses in relation to cancer research, with some focusing on specific tumours [25–27]; others have looked at cancer research activity within a particular country [28–30] or more widely across larger geographic regions and continents [31, 32]. There has, as yet, been no global bibliometric analysis of rural cancer research scientific publications. This research aimed to understand: (1) **publication trends** in rural cancer research over time; (2) **where** (countries) rural cancer research is being conducted?; (3) **who** (institutions) is conducting rural cancer research, as well as the extent and **patterns of collaboration** (between countries) with other rural cancer researchers?; (4) **which journals** are publishing rural cancer research?

## Methods

### Study design

Bibliometric methods were used to provide a general and comprehensive synopsis of activity in rural cancer research [24]. This study adhered to the Guideline for Reporting Bibliometric Reviews of the Biomedical Literature (BIBLIO) [33].

### Data sources

Web of Science and Scopus were used to deliver this analysis. These sources were chosen to provide an extensive range of literature in medical, health, nursing, public health, and social sciences. Web of Science is widely used in bibliometric studies [25] and covers a significant number of academic journals across the medical and social sciences. Similar to Web of Science, Scopus encompasses peer-reviewed literature from the medical and social sciences, as well as the arts and humanities. Databases such as PubMed, CINAHL, and PsycINFO were excluded, as they lack metadata concerning corresponding authors and citations, both of which relate directly to the research aims mentioned above.

### Search strategy

A topic search targeting specific keywords was conducted in Web of Science using the following syntax: TS = (Cancer AND Rural). The search results were downloaded in BibTeX file format. A title, abstract, and keyword search was performed in Scopus: (TITLE-ABS-KEY (rural)) AND (TITLE-ABS-KEY (cancer)). Scopus results were downloaded in CSV file format. Topic, title, abstract, and keyword searches suggest that the studies explicitly focused on rurality and cancer. No date restrictions were set. Given the global nature of the bibliometric analysis, articles published in non-English languages were included, although all articles in the dataset had at least titles, abstracts and keywords in English. We only included academic research literature that was published in peer-reviewed journals. Grey and non-academic literature were excluded. Searches were conducted on 25th February 2025.

### Data preparation and analysis

Bibliometric records from both databases were imported into R 4.4.2 (https://www.r-project.org/) and processed using the *bibliometrix* package [34, 35]. Records were converted into standardised data frames, filtered to include only peer-reviewed scientific articles, and cleaned to remove incomplete or anonymous entries. Duplicates were removed, and affiliations were matched across sources. The final dataset retained key metadata: publication details, author affiliations, content descriptors, and citation counts.

Descriptive and network analyses were conducted using *bibliometrix* and VOSviewer. Scientific output, citation metrics, and collaboration patterns were assessed, including country-level productivity and citation impact (adjusted for publication age). Thematic and factorial analyses of author keywords were performed to identify research trends and conceptual structures. Full methodological details are available in Supplementary Information (S1).

## Results

The bibliometric analysis was conducted on 15,722 documents published from inception (1904)- February 2025. Data were complete for authors, document type, journal, publication year, title and total citations. Ninety five percent of the articles were published in English (*n *= 15,005). An overview of data completeness and the included documents can be found at Supplementary Information (S2). The mean age of documents was 10.6 years (SD = 10.24) and mean number of citations per document was 25.5 (SD = 131.39). The mean citations per year per document was 2.2 (SD = 12.85). The annual growth rate in publications was 4.6%. A log-linear regression revealed a highly significant positive trend in annual scientific publications, with a compound annual growth rate (CAGR) of 8.99% (β1 = 0.086, *p *< 0.001).

**Fig. 1:**
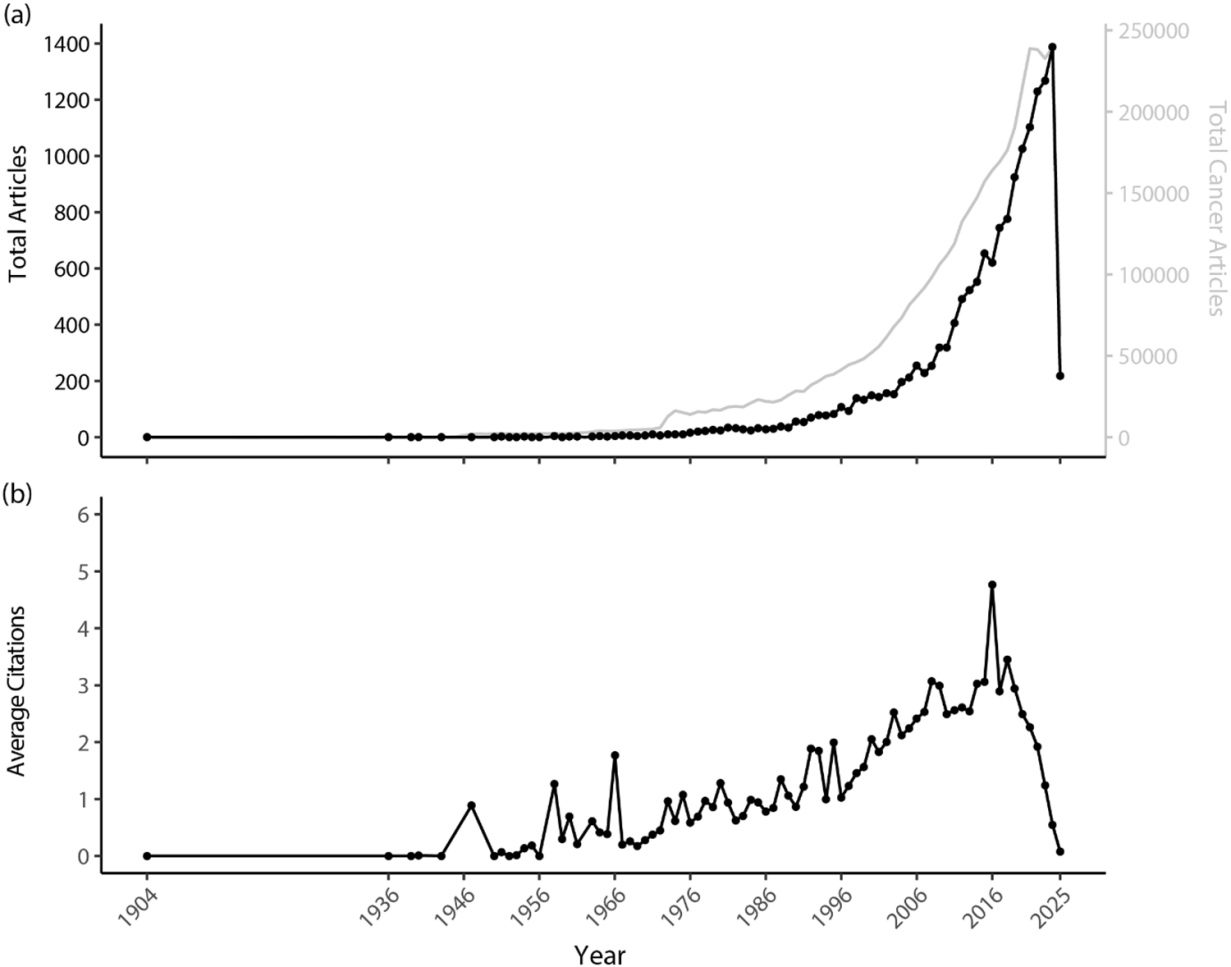
Publications trends of rural cancer research, with **a** showing the annual production of rural cancer articles (black) and general cancer articles (grey; with data being extracted from Scopus), and **b** the average article citations per year. Note: Average article citations per year reflects the annual citation rate of articles, normalized by their age, which typically results in lower values for more recent publications due to citation lag. (Color figure online)

There is a clear and ongoing escalation in the publication rate starting in 2006. Similarly, the average article citations per year have increased over time, peaking in 2016 at an average of five citations per year (Fig. 1). Subsequently, this falls to the present value which is likely due to an inherent lag in the citation process as more recent publications have less time to accrue citations compared to older articles.

**Fig. 2:**
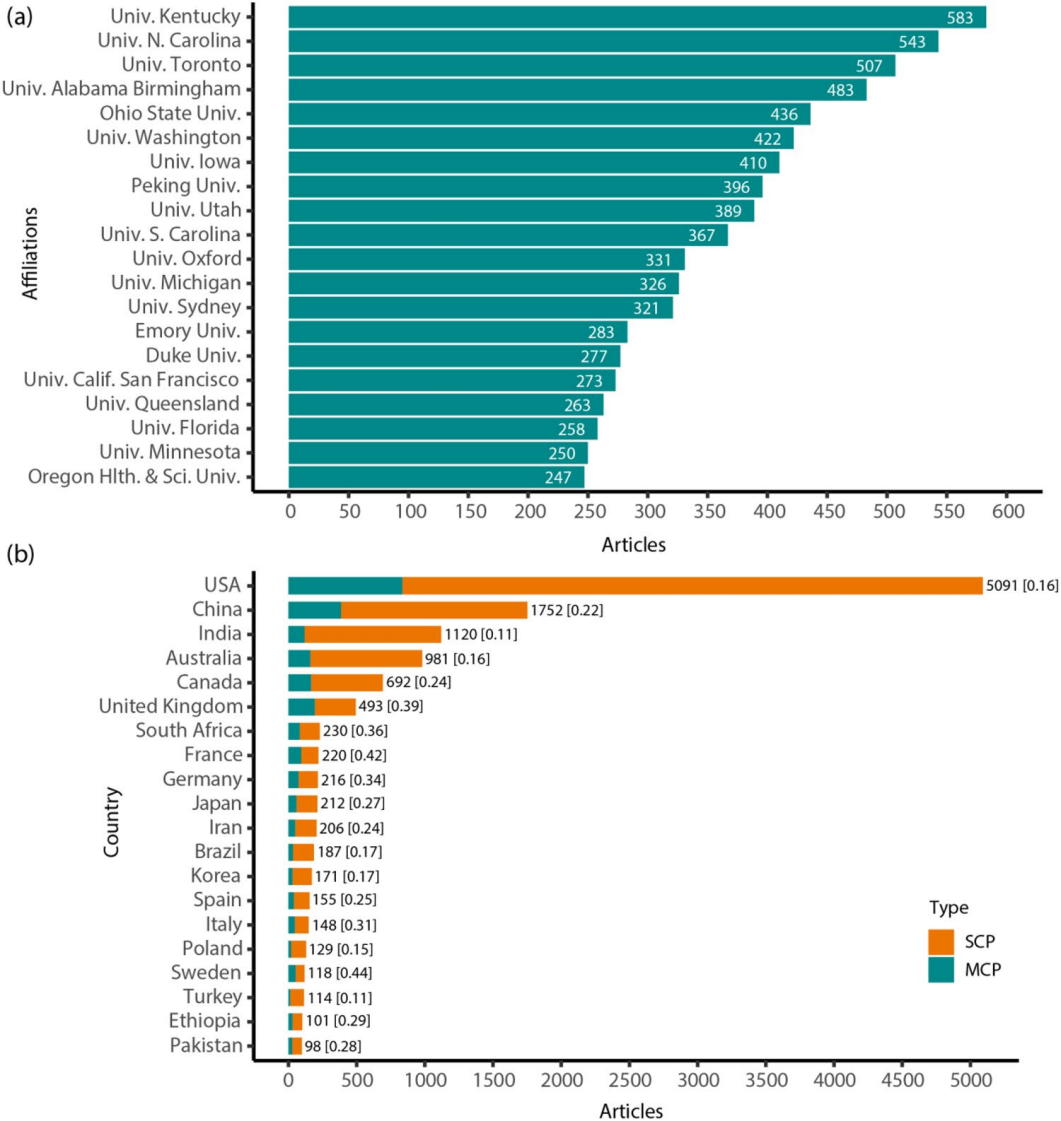
Top 20 most productive **a** institutions using all co-authors of a publication, and **b** countries by corresponding author. Note: Showing the total number of publications at the end of each column with the MCP (multi country publication) to total publications ratio in square brackets. The higher the ratio the more collaboration between this country and others. SCP (Single country publication),

The productive institutions (Fig. 2a) were predominantly located in North America—the top three were the University of Kentucky, University of North Carolina and the University of Toronto, with > 500 articles. The majority of the most productive institutions were based in the USA (*n *= 15) with only two in Australia (University of Sydney, University of Queensland), one in Canada (University of Toronto), one in China (Peking University) and one in the UK (University of Oxford).

**Fig. 3:**
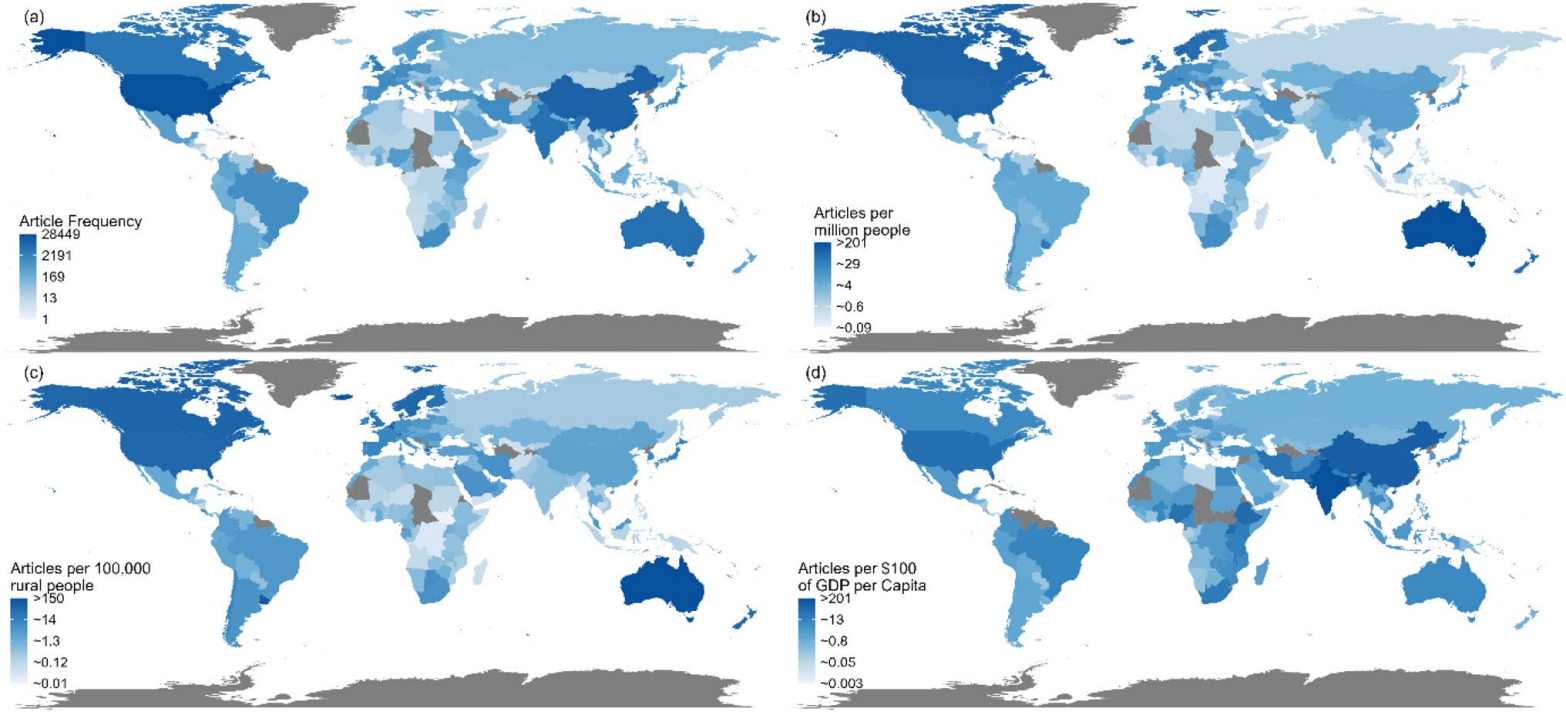
World maps of log transformed cumulative production with countries of all co-authors. **a** total articles, and articles adjusted for **b** population size, **c** rural population size, **d** GDP per Capita. Note: Darker blue colours denote higher productivity while regions with light blue have lower publication productivity. Areas in grey denotes no data available or no activity. (Color figure online)

The top six countries by corresponding author were USA, China, India, Australia, Canada and the UK (Fig. 2b). The remaining fourteen were from Europe (*n *= 6), East Asia (*n *= 2), Western Asia (*n *= 1), South Asia (*n *= 1), Africa (*n *= 2), South America (*n *= 1) and Turkey (*n *= 1), which straddles Southeast Europe and West Asia. The distribution of publications on the world map, using all co-authors, are shown in Fig. 3. Similar to only using the corresponding authors’ country affiliation, authors affiliated with the USA (*n *= 28,437), China (*n *= 8,928), Australia (*n *= 5,353), India (*n *= 4,996) and Canada (*n *= 4,048) are involved the most in rural cancer research articles. Adjusting for total population revealed Australia (*n *= 201), Norway (*n *= 79), Iceland (*n *= 76), Denmark (*n *= 63), Sweden (*n *= 58), and New Zealand (*n *= 56) as leading countries in publications per million people. This *per capita* output stood in contrast to lower figures from India (*n *= 3) and China (*n *= 8), while the USA (*n *= 85) and Canada (*n *= 101) “continued to make substantial contributions on a per-person basis” maintained considerable contributions in this normalized view. The map adjusted by rural population size exhibited trends similar to the total population adjustment, though it highlighted Australia (*n *= 150), Iceland (*n *= 128), Belgium (*n *= 99), and Uruguay (*n *= 95) as leaders in publications per hundred thousand people, with the USA, Canada, and Nordic countries closely following. Finally, adjusting for GDP per capita yielded a distinct distribution, wherein European countries generally converged in their research output. Notably, India (*n *= 201) and China (*n *= 88) registered the highest publication records (all expressed as publications per $100 of GDP), followed by the African nations Ethiopia (*n *= 45) and Uganda (*n *= 40), and then Pakistan (*n *= 34) and the USA (*n *= 34).

Table 1 reports on country citation data using the corresponding author. The USA, China, Australia, UK and Canada lead in the highest number of citations. In terms of average citations, the five highest per country were Denmark 43.14 (SD = 47.92), UK 41.48 (SD = 67.13), Sweden 41.44 (SD = 89.35), Greece 40.35 (SD = 117.46) and France 36.38 (SD = 103.47), indicating strong per-paper impact. China also has a high average citation per country with 34.93 (SD = 359.52) and per year with 4.74 (SD = 40.87) but with a high standard deviation, indicating a wide citation range and extreme variability. The countries with the lowest median citations per year were India with 0.67 (IQR = 0.00–1.67), Turkey 0.82 (IQR = 0.13–2.23), Japan 0.90 (IQR = 0.33–2.03) and Brazil 1.00 (IQR = 0.16–2.00), indicating relatively low impact per year despite publishing rural cancer articles and in the case of India and Brazil having large populations with a substantial proportion living in rural settings.

**Table 1:** Country citation data using the corresponding author

Country	Total articles	Total citations	Average citations per Country (SD)	Average citations per year (SD)	Median citations per year (IQR)
USA	5,091	141,634	27.82 (61.90)	2.77 (4.92)	1.54 (0.66–3.14)
China	1,752	61,202	34.93 (359.52)	4.74 (40.87)	1.60 (0.50–3.75)
Australia	981	20,502	20.90 (32.96)	2.18 (2.86)	1.40 (0.62–2.64)
UK	493	20,452	41.48 (67.13)	3.55 (5.84)	1.75 (0.77–3.82)
Canada	692	17,295	24.99 (65.88)	2.72 (7.05)	1.42 (0.63–2.84)
India	1,120	16,391	14.63 (43.50)	1.90 (5.85)	0.67 (0.00–1.67)
France	220	8,003	36.38 (103.47)	2.68 (6.33)	1.56 (0.40–2.59)
South Africa	230	5,612	24.40 (71.93)	2.07 (5.02)	1.00 (0.42–2.13)
Sweden	118	4,890	41.44 (89.35)	3.25 (6.58)	1.68 (0.85–3.74)
Japan	212	4,311	20.33 (33.74)	1.60 (2.11)	0.90 (0.33–2.03)
Italy	148	4,103	27.72 (45.48)	2.05 (3.22)	1.00 (0.50–2.00)
Germany	216	3,756	17.39 (26.13)	1.95 (2.34)	1.15 (0.44–2.68)
Iran	206	3,524	17.11 (31.08)	2.48 (4.62)	1.05 (0.33–2.79)
Denmark	78	3,365	43.14 (47.92)	3.16 (3.48)	1.75 (0.95–4.17)
Greece	78	3,147	40.35 (117.46)	2.89 (5.99)	1.39 (0.51–2.80)
Spain	155	2,645	17.06 (25.34)	1.80 (2.83)	1.00 (0.33–2.00)
Norway	92	2,504	27.22 (41.56)	1.94 (2.62)	1.05 (0.58–2.20)
Brazil	187	2,458	13.14 (20.75)	1.65 (2.30)	1.00 (0.16–2.00)
Korea	171	2,261	13.22 (17.75)	1.81 (1.97)	1.13 (0.50–2.28)
Turkey	114	2,127	18.66 (44.36)	1.75 (2.87)	0.82 (0.13–2.23)

**Note:** Countries ordered by total citations

**Fig. 4:**
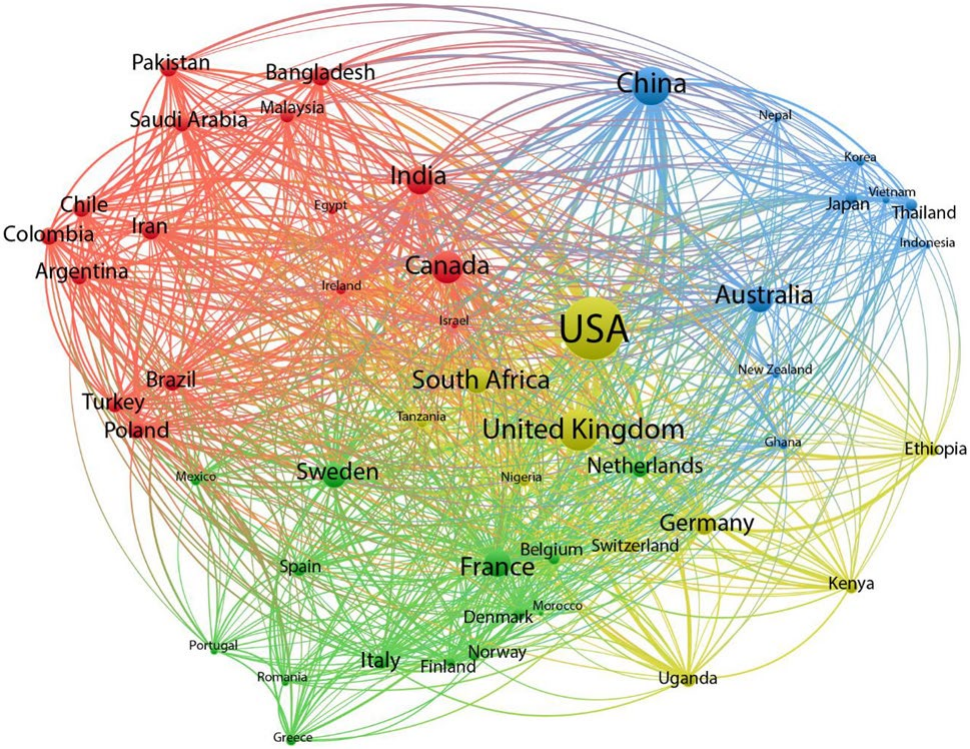
Weighted collaboration network of the top 50 most productive countries. Note: The size of the nodes (countries) corresponds to their production, and the edge thickness (i.e., weighted connections between nodes) corresponds to their co-occurrence in the publications. Clusters (group of countries that are collaborating more with each other compared to other countries in the network, i.e., distinct subgroup) are shown in the same colour. (Color figure online)

Figure 4 shows collaborative links among countries working on rural cancer research. Countries who published at least 150 articles (using affiliations from all co-authors) were included in the network, resulting in a 50-country network. Centrally placed countries in the graph, such as the USA, UK and Canada, have a high volume of collaborative research publications with other nations, and can be seen as key players in the global research landscape by connecting regions. All three of these nations are English speaking countries with Canada being bilingual (English and French its official languages). China on the other hand, placed more on the periphery of the network, shows also a high volume of research outputs, leading to their large node size; however, it has fewer collaborations with other countries.

Overall, the collaboration network is presented in 4 different clusters, which are shown in different colours. Clusters refer to groups of countries that are collaborating more closely with each other than with countries outside of the cluster, essentially forming a distinct subgroup within the larger collaboration network. One cluster is centred around the USA and UK, including countries such as Germany, The Netherlands and countries in Africa such as South Africa. A second cluster has Canada as the most productive country, with a strong connection to India. The third cluster contains mainly European countries, including France, Sweden, Spain and Belgium. The final cluster contains China and Australia, as the countries producing the most publications in collaboration with several other Asian countries. The thickness of the edges between countries represents the strengths of the research collaboration between them, showing for instance strong collaboration ties between the USA and China, UK and India.

**Fig. 5:**
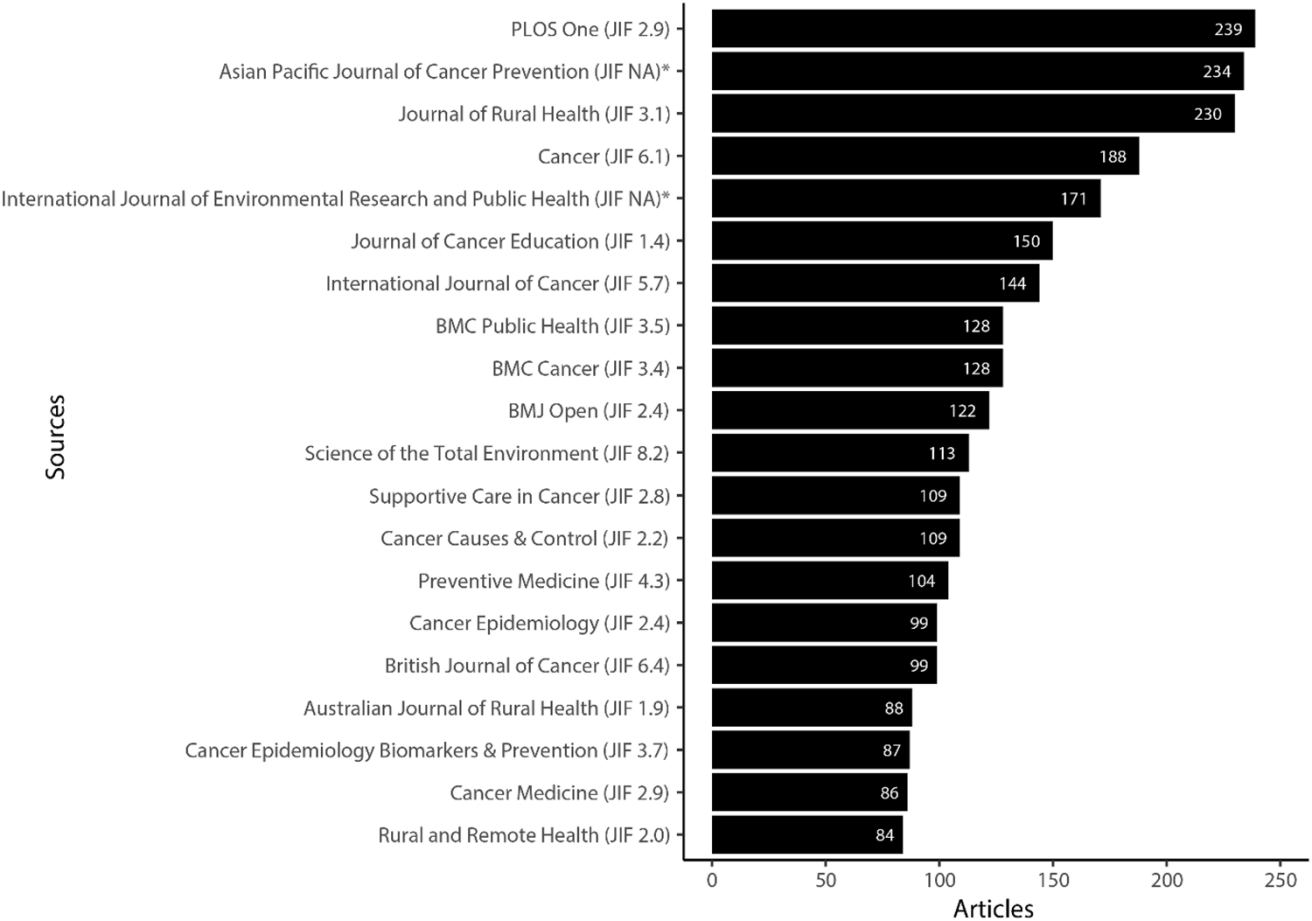
Top 20 journals publishing rural cancer research. Note: JIF denotes journal impact factor from 2023, taken from the Journal Citation Reports (JCR) database. *No recent JIF available for these journals

Figure 5 shows that the top five journals publishing rural cancer research are PLOS One (*n *= 239), Asian Pacific Journal of Cancer Prevention (*n *= 234), Journal of Rural Health (*n *= 230), Cancer (*n *= 188) and International Journal of Environmental Research and Public Health (*n *= 171). Across the top 20 journals publishing rural cancer research, the publications fall into the following categories, cancer-specific (*n *= 11), rural health (*n *= 3), public health (*n *= 3), general medical or health journal (*n *= 2) and environmental science (*n *= 1).

**Fig. 6:**
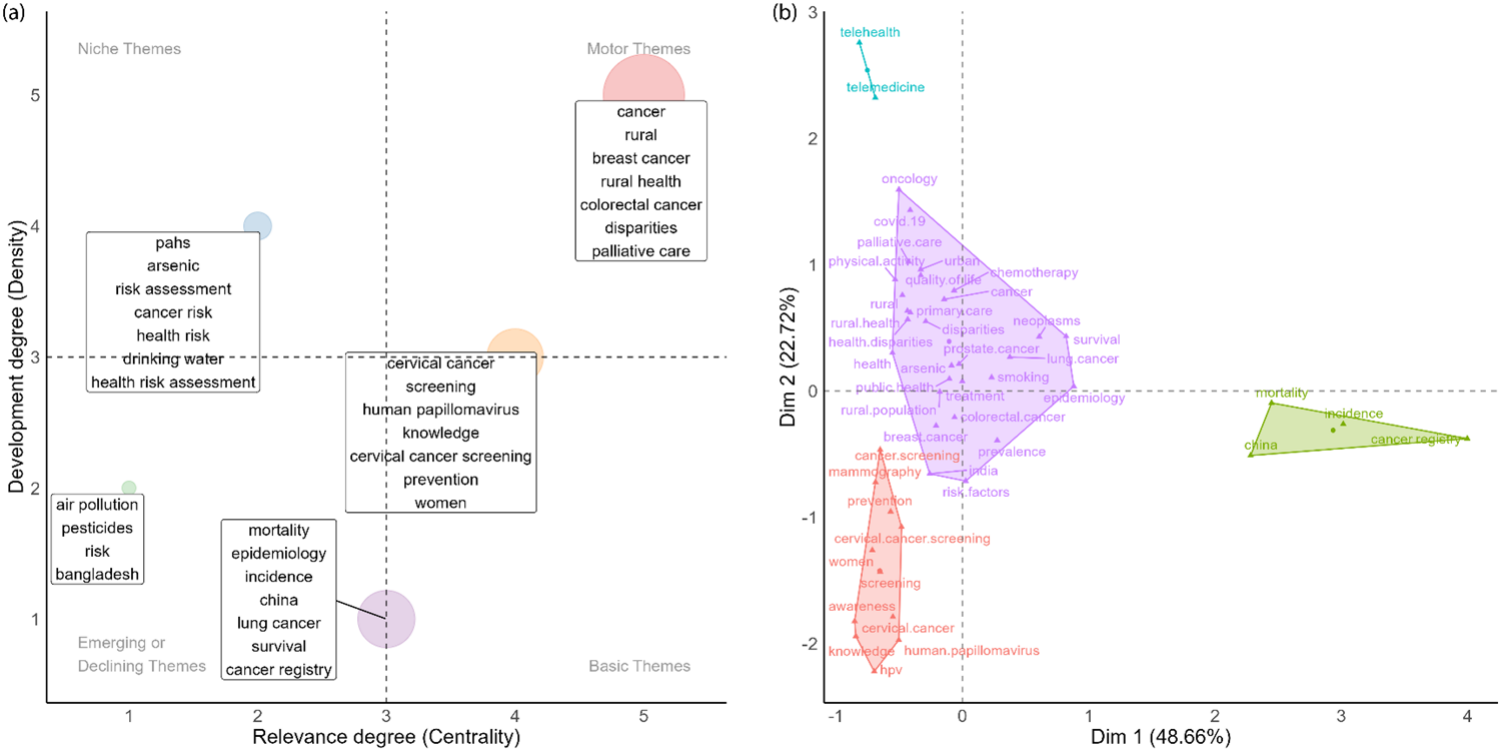
Conceptual structure of author keywords in rural cancer research. **a** Thematic map, visualizing distinct thematic clusters within a strategic diagram. **b** Factorial analysis, illustrating the underlying conceptual dimensions structuring keyword relationships. Note: (a) Thematic Map: Node size is proportional to keyword frequency, indicating the overall prominence of keywords within that cluster. Colours represent the distinct thematic clusters (identified by the Walktrap cluster algorithm). Map quadrants are defined by thematic centrality and categorised as motor themes (upper-right), niche themes (upper-left), basic themes (lower-right), and emerging or declining themes (lower-left). (b) Factorial Analysis: The X and Y axes represent the two principal dimensions from the Multiple Correspondence Analysis (MCA) that explain [X]% and [Y]% of total variance. Colours denote distinct conceptual clusters of keywords identified within this factorial space. The position of keywords and clusters on the dimensions reflects their conceptual meaning and interrelationships within the mapped space. (Color figure online)

The conceptual structure of rural cancer research shown through a thematic map and factorial analysis of author keywords in Fig. 6 indicates the core themes and evolving areas within the scientific field. Panel (a), the thematic map, organises keyword clusters based on their centrality (relevance) and density (development). Motor themes (highly developed and relevant) like rural health, disparities, breast cancer and colorectal cancer are well-established. The basic themes (highly relevant but less well developed) include cervical cancer, screening, HPV, prevention and women, these are central issues that merit further research activity. Niche themes (of lower relevance but with high development) such as arsenic, health risk assessment and cancer risk are both well developed and peripheral, relating to specific methodological or geographic contexts. Emerging or declining themes (with limited relevance and less well developed) such as air pollution, pesticides and Bangladesh show new research directions or declining interest. Panel (b), the factorial analysis, shows the co-occurrence of author keywords. Rural health disparities, cancer types and outcomes represent a dominant cluster. Cervical cancer and HPV is a distinct cluster, highlighting an emerging but underdeveloped area. Another isolated cluster on telehealth and telemedicine highlights a significant and nascent area in rural cancer research, reflecting how the world has changed post-pandemic. Finally, an epidemiology focused cluster on incidence, mortality and China indicates regionally specific research activity.

## Discussion

Our study is the first bibliometric analysis of the global state of rural cancer research spanning multiple decades, offering a long-term view of rural cancer research activity. The study has several strengths which we wish to acknowledge. Firstly, it is based on a large dataset (15,722 documents) from two well-established, credible and widely used academic databases (Scopus and Web of Science) with excellent metadata to allow for meaningful trend detection. Secondly, our study was global, incorporating publications from a wide range of countries, regions and continents. This improves our understanding of the rural cancer research landscape on a global scale and limits geographic bias. Thirdly, the use of a long-term analytical range (1904–2025) offers over a century’s worth of insight into how research activity, collaboration and thematic priorities in rural cancer research have evolved over time. Finally, the use of weighted collaboration networks, thematic clustering and factorial analysis techniques, allows for a more detailed analysis of collaborations, emerging trends and structures within the field. Together, these all provide a rigorous and robust overview of rural cancer research’s evolution and current state.

The findings demonstrated that the annual production of rural cancer research articles broadly mirrors the overall growth in cancer research, showing a consistent upward trend. This is a positive development, especially considering the projected global cancer burden, with over 35 million new cases expected by 2050, an increase of 77% from current levels [36]. However, while publication and citation rates have increased, much of this growth appears concentrated within a small number of institutions and countries. This concentration raises concerns about representativeness. A substantial proportion of the global population lives in rural areas [14], many of which are outside these high-output countries. It is essential for research efforts to align with the geographic distribution of need, particularly given the well-documented health inequities associated with rurality. Rural populations face distinct challenges across the entire cancer care continuum from prevention and early detection to diagnosis, treatment, survivorship, and mortality [4–7, 21]. Therefore, there is a critical need for geographically distributed, context-specific research that encompasses all cancer types and research domains.

The average age range of 10.6 years for documents indicates that the scientific literature is representative of both legacy and recent works. Average citations per document show moderate academic impact in the field. The value of 25.5 citations per year per document shows consistent citation performance, although a small number of highly cited documents could be inflating the mean. The annual growth rate of 4.6% shows sustained growth, with a rapid growth since 2006. Furthermore, the log-linear regression revealed a highly significant positive trend in annual scientific publications, with a CAGR of 8.99%.

Geographically, the top six countries by corresponding author in terms of the number of papers published were the USA, China, Australia, India, Canada and UK. With the exception of the UK, this dominance may reflect national cancer policies and priorities that emphasise broader rural health inequalities. Indeed, the USA [37], China [38], Australia [39], and Canada [40] all acknowledge to varying degrees the challenges of rural and remote living in their most recent national cancer plans.

Findings indicate that the UK is becoming a significant contributor in this field with an increasing volume of rural cancer research. However, when it comes to UK cancer policy, rurality has long been neglected and there have been recent calls for policymakers to urgently recognise the importance of “place” in cancer control and planning [41]. In contrast, much of Europe falls behind, despite having extensive rural landscapes and more than a quarter of its population living in these areas [14]. This disparity offers a valuable opportunity for the oncology and rural health research community, to concentrate efforts on rural cancer issues and attract investments in rural cancer research throughout Europe. A particular focus for rural cancer research could be central and eastern European countries, who have a high proportion of rural people [14] and where existing evidence highlights the widening gap in cancer research activity, capacity and outcomes, compared to the rest of Europe [42]. It should be noted that our adjusted analyses on productivity by country highlights that raw publication figures can mask important contributions from countries with diverse demographic profiles and economic capacities, indicating nations that are particularly productive or strategically focused on rural cancer research. At the same time, countries such as the USA and Australia still dominate in overall numbers after adjustment for population size and rural population. Their dominance is diminished on adjustment for GDP, where India stands out and is joined to a lesser degree by some central Asian and African nations.

Our analysis on country citations shows that while USA, China, Australia, UK and Canada lead in overall citations, European countries such as Denmark, Sweden and the UK have above average citations per-paper, showing strong impact on a per publication basis. China’s high citation count is likely influenced by a smaller number of highly cited and lower impact publications, given the high standard deviation observed.

The most productive institutions were mainly located in North America. This North American dominance suggests established research infrastructure, funding and institutional support for rural cancer research, with higher research capacity and output from larger universities [43]. Research on scientific productivity shows that North American and other anglophone countries are not just prolific in terms of scientific output, but also diverse in terms of content [44]. So the dominance in rural cancer research as an emerging field is not surprising. Less developed nations tend to only be competitive in scientific fields where many other nations are present [44]. The emerging presence of both China and India highlights their increasing influence in rural cancer research which could be driven by investment in science and education [45, 46] as well as both having large rural populations [14].

While place-based inequalities require tailored, context-specific solutions, much can be gained from cross-institutional and cross-national collaboration. Our findings indicate that such partnerships are already occurring, and we strongly encourage the expansion of collaborative networks, particularly those that include underrepresented regions and institutions. The patterns of collaboration suggest that rural cancer research activity is shaped by regional and geopolitical collaborations that are likely influenced by shared health priorities, historical ties or funding mechanisms. The presence of intercontinental links (e.g., UK and India, USA and China) show that global partnerships are driving rural cancer research activity. Still, some of the more distinct clusters could indicate variation in research priorities or limited access to collaborative networks. Our results reinforce opportunities for new global collaborations and capacity building in rural cancer research to strengthen local research ecosystems by identifying countries with comparatively limited research output.

The findings presented here, highlight that rural cancer research is being published across a wide range of journals, with strong representation in both cancer-specific and interdisciplinary journals. Whilst cancer journals are the highest in terms of outputs, not surprisingly rural health and public health journals are also key contributors to the dissemination of rural cancer research.

The thematic analysis identified several recurring topics, but notably lacked emphasis on early diagnosis, a vital component in improving cancer outcomes, particularly in rural settings where delayed presentation and access barriers are common. Future research agendas should aim for comprehensive thematic coverage, ensuring that underserved aspects such as early diagnosis care are not overlooked. The analysis of journals and thematic analysis suggests that some selected articles are likely to be identifying biomakers/aetiological factors. Whilst important, these might reasonably be considered a distinct group whose implications for policy lie in public health and cancer prevention, as opposed to the delivery of cancer services for rural populations. Beyond the niche and emerging themes, there is a consistent focus across the literature on cancer diagnostic pathways and cancer screening. Further focus on specific cancer diagnoses (colorectal, breast and cervical cancer) and the delivery of palliative care in rural settings is also seen, highlighting areas of current strength in the published literature. Breast, colorectal, and lung cancer all appeared in our thematic map suggesting that rural cancer research is currently representative of more common cancers and that rarer cancers are potentially neglected. Further research should quantify the types of cancer that are being studied in rural areas with a view to informing subsequent research activity on lesser-studied tumour types. Rarer cancers can be particularly challenging for rural communities due to the urgent need for specialised care, timely diagnosis, and access to trials or treatments that are usually located in more densely populated urban areas [5].

Finally, we must be mindful of intersectionality in understanding rural health inequities. Rurality intersects with other protected characteristics, such as ethnicity, disability, age, and socioeconomic status, compounding disadvantage. Future research and policy must adopt an intersectional approach to ensure equitable cancer outcomes for all rural populations, particularly those facing multiple, overlapping forms of marginalisation.

### Limitations

A key challenge in rural cancer research is the lack of a global definition of rurality [47]. Conceptually, ‘rural’ can be highly subjective, is amenable to cultural and socio-economic factors, and varies across nations and regions. Problems and priorities will differ across geographies. This inconsistency makes cross-country comparisons and the transferability of research findings challenging. Future rural cancer research should aim to establish transparent and, where possible, standardised definitions of rurality, to facilitate the comparability and applicability of research findings.

While the term ‘remote’ is widely used in countries such as Australia and Scotland, we excluded it from our search terms, as it risked inflating the number of retrieved articles with irrelevant literature (e.g., ‘remote consultation’) since this does not imply that the studies were explicitly set in a rural/remote area. Remoteness is well contextualised within the wider rural health extant literature; therefore, it is likely that searching for rural alone would still retrieve these papers. Bibliometric analysis typically employs a broad search strategy to account for the wide nature of scientific activity within a field, when compared to a more focused systematic review [24]. It is thought that if the terms Cancer and Rural appear within the title, abstract, or article keywords, then it would be explicit enough that the retrieved record has some degree of focus on cancer and rurality.

The inability to search databases such as PubMed is a considerable limitation which we wish to highlight. For bibliometric methods in biomedical, oncology and health-related disciplines to advance, bespoke databases such as PubMed will need to improve in terms of the metadata (e.g., missing citation data, author and institutional disambiguation) that they record, so researchers do not need to rely solely on databases such as Scopus and Web of Science. Therefore, there is a risk that some of the key clinical literature could have been neglected in our bibliometric analysis. For topic and trend bibliometric analyses PubMed may still be suitable, however, for network and citation-based bibliometrics like ours, urgent metadata improvements are necessary before it can be fully utilised.

A notable omission from our analysis was the inclusion of cancer research that focuses on coastal areas. Much like rural areas, coastal areas can suffer from reduced access to cancer services, workforce issues and poor transport infrastructure [48, 49]. At the same time, the nuances and differences between rural and coastal communities need to be recognised and they should not be treated as one and the same [41]. Coastal communities can experience high levels of socio-economic deprivation, transient and seasonal populations, environmental threats and social immobility [50, 51]. They are geographically distinct in that they can be rural or urban. Despite recognition from high-level UK policymakers [52] around the health and social challenges faced by coastal communities [50], this is a relatively new area of academic inquiry when it comes to the study of “place” and cancer and there is a lack of international high-quality evidence in this area. At present, the coastal challenge appears to be one that is somewhat unique to the UK context. Further research on the impact of residing in coastal areas and cancer outcomes is necessary. We welcome UK-based and international studies that specifically explore the intersection of coastal living with high levels of socio-economic deprivation, as it is likely that accessing and engaging with cancer care will be extremely challenging for these communities.

While institutional affiliations provide insight into where research is produced, they do not necessarily reflect the location of the populations studied. For example, a study led by a U.S.-based institution may focus on rural populations in other countries. Additionally, this analysis did not assess the quality or methodological rigour of the included publications. Some articles may reference rural settings without directly addressing rural health outcomes, which could affect the relevance and specificity of the findings.

## Conclusions

This bibliometric analysis was the first to provide a global overview of rural cancer research activity, showing a positive growth in publication activity, increasing international collaborations and emerging subject-specific diversity. However, the concentration of research in mostly high-income settings indicates an imbalance in global representation. To advance equity in cancer care and outcomes, future research efforts need to prioritise context-specific research and expand collaborations to underrepresented regions, where capacity and infrastructure is limited. These findings can guide future funding priorities as well as capacity building and new global academic partnerships. Intersectional rural cancer research that recognises the complexity and nuances of rural communities is urgently required. Addressing some of the gaps and challenges such as a lack of early diagnosis research, as well as insuring inclusion of all aspects of the cancer pathway in research, clearer definitions of rurality, will be essential in driving academic productivity and impact of rural cancer research going forward.

## Supplementary Information

Below is the link to the electronic supplementary material.

Supplementary file1 (DOCX 55 KB)

Supplementary file2 (DOCX 16 KB)

## Data Availability

The data that support the findings of this study are available from the corresponding author (DN), upon reasonable request.
