# Supplementary material for: Mapping the landscape of rural cancer research: a global bibliometric analysis
Source: Cancer Causes Control. 2025 Dec 27;37(1):5. doi: 10.1007/s10552-025-02086-0 (PMC12743660; doi:10.1007/s10552-025-02086-0)
Supplement: Supplementary file 1 — Supplementary file1 (DOCX 55 KB) [file 10552_2025_2086_MOESM1_ESM.docx]

**Supplementary Information 1 (S1)**

**Detailed Data Preparation and Analysis**

Results from both databases were imported into R 4.4.2 (<https://www.r-project.org/>) and converted into bibliographic data frames using the *convert2df* function in the *bibliometrix* package.^1, 2^ Cases in the data frame correspond to articles/manuscripts and variables to their metadata (e.g. authors’ names, title, abstract, keywords, etc.) named using standard Clarivate Analytics WoS Field Tag codify. Further field tags were extracted using the *metaTagExtraction* function to obtain the country affiliation of all co-authors and the corresponding author, as well as the university affiliation for each co-author. Both data frames were separately restricted to only contain peer-reviewed scientific articles (using the “DT” field tag; see Tables 1 and 2 in the supplementary material for included document types). Documents containing only anonymous authors^3^ were removed from the Web of Science data frame, and field tags containing author affiliations were cleaned by removing (leaving as missing) entries containing only addresses, initials or a label stating corresponding author. Similarly, records with no authors and affiliations were removed from the Scopus data frame (see Figure S1 for data preparation flow chart).

Following this, both data frames were merged and duplicate records removed using the *mergeDbSources* function, which identifies common tag fields and duplicated records (i.e. author names, title, doi, etc.). Custom R code was used to match up affiliations between both data frames, specifically abbreviating all Scopus affiliations following the Web of Science address abbreviations found [online](https://support.clarivate.com/ScientificandAcademicResearch/s/article/Web-of-Science-Address-abbreviations?language=en_US). Manual cleaning of some country names was further necessary to correctly plot the bibliometric world map data.

Finally, unused variables (field tags) were removed, and the final dataset contained only the following metadata used in the analysis: (a) document information, including publication year, journal title, and DOI; (b) author information, including authors’ name and affiliations (institutions and countries); (c) content information, including title, abstract, and authors’ keywords, (d) citation information detailing the total number of citations of each record.

**
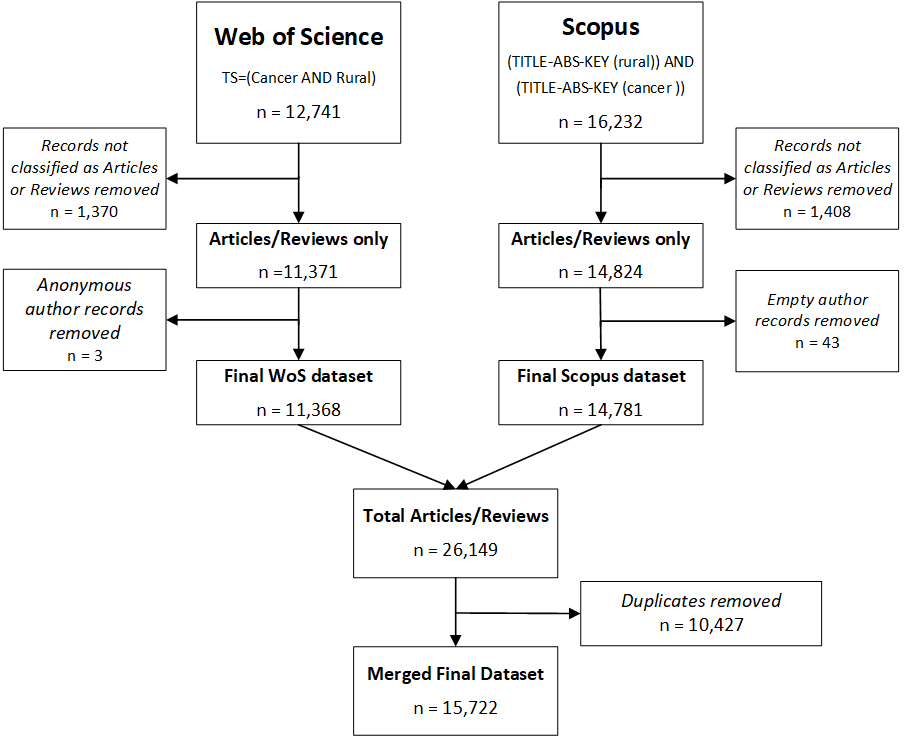
**

**Figure S1: Flowchart of data preparation for the rural cancer research bibliometric analysis**

**Analysis**

All analyses and visual illustrations were conducted in R 4.4.2. The dataset was assessed for completeness, and an overview of the main information was extracted using the *bibliometrix* functions *missingData* and *summary*. The *biblioAnalysis* function was employed for descriptive analyses of (1) annual scientific production and citations, (2) the most productive countries and institutions, and (3) top sources/journals publishing rural cancer research. The country of the corresponding author was used to identify the top 20 most productive countries and further assess the ratio of multiple country publications (MCP; at least one co-author was affiliated with a country other than that of the corresponding author) to total publications. Furthermore, world maps illustrating the total production of countries, based on all authors of a publication, were created using the *map_data* function from the *ggplot2* package, with data adjusted for total population, rural population, and GDP per capita using 2023 data from the World Bank website.

Quality of publications across countries was assessed using citation data. To provide a measure of the overall citation volume and its variability, the average (SD) of the total citation counts were calculated for each country. Furthermore, citation data was adjusted for publication age. For each record, the number of years since publication was calculated based on the current year of 2025. For records with a publication year prior to 2025, the years since publication were determined by subtracting the publication year from 2025. For records with a publication year of 2025, a fractional year value of 2/12 was used to represent the time elapsed since publication until the database search. The citations per year were calculated by dividing the total citations by the years since publication. These per-record yearly citation rates were then aggregated by country. To summarize the central tendency and dispersion of these rates for each country, the median and interquartile range (IQR) were calculated. Additionally, the mean and standard deviation (SD) of the per-record yearly citation rates were also determined for each country.

A country collaboration network of the 50 most productive countries was computed using the *biblioNetwork* function and visualised using VOSviewer software^4^ version 1.6.20. The size of the nodes (countries) in the network graph corresponds to their occurrence in the dataset and edge thickness (i.e. weighted connections between nodes) correspond to their co-occurrence in the records of the dataset. The network was normalised using a probability-based measure called association strength. This measure is dividing the observed number of co-occurrences over the expected numbers of co-occurrences.^5^ Walktrap clustering algorithms were used to detect clusters^6^ and they are shown in the same colour in the graph.

A thematic analysis was conducted using author keywords, arranging the themes according to their impact and centrality.^7-9^ The top 250 most frequent terms were included, with an additional restriction that they had to occur at least 3 times (standard value used in the package) in the dataset. The thematic map is organised by four quadrants: niche (top left) which consist of unique research areas representing a narrower topic that has emerged from basic themes, motor (top right), these are well-established and extensively studies themes. They represent fundamental concepts, theories and methodologies of rural cancer research, emerging or declining (bottom left) which relate to areas of research gaining prominence as well as areas of future direction, trends or declining interest or relevance, and basic themes (bottom right), which are broader areas of research that build upon the motor theme. Finally, to explore the underlying structure and relationships among author keywords, a factorial analysis using multiple correspondence analysis (MCA) was performed.^2^ MCA is a multivariate statistical technique that reduces the dimensionality of the keyword data to visualize the associations between different keywords in a low-dimensional space, allowing for easier interpretation of complex keyword relationships.^10^ This technique allows for the identification of clusters of keywords that tend to co-occur in publications. Only words that appeared at least 100 times in our dataset were included in the analysis.

**References**

**1.** Aria M, Cuccurullo C, Aria MM. Package ‘bibliometrix’: CRAN. <https://cran>. rproject. org/web/packages/bibliometrix/bibliometrix. pdf; 2022.

**2.** Aria M, Cuccurullo C. bibliometrix: An R-tool for comprehensive science mapping analysis. *Journal of informetrics.* 2017;11(4):959-975.

**3.** Shamsi A, Silva RC, Wang T, Raju NV, Santos-d’Amorim K. A grey zone for bibliometrics: publications indexed in Web of Science as anonymous. *Scientometrics.* 2022;127(10):5989-6009.

**4.** Van Eck N, Waltman L. Software survey: VOSviewer, a computer program for bibliometric mapping. *scientometrics.* 2010;84(2):523-538.

**5.** Eck NJv, Waltman L. How to normalize cooccurrence data? An analysis of some well‐known similarity measures. *Journal of the American society for information science and technology.* 2009;60(8):1635-1651.

**6.** Lancichinetti A, Fortunato S. Community detection algorithms: a comparative analysis. *Physical Review E—Statistical, Nonlinear, and Soft Matter Physics.* 2009;80(5):056117.

**7.** Donthu N, Kumar S, Mukherjee D, Pandey N, Lim WM. How to conduct a bibliometric analysis: An overview and guidelines. *Journal of business research.* 2021;133:285-296.

**8.** Chang Y-W, Huang M-H, Lin C-W. Evolution of research subjects in library and information science based on keyword, bibliographical coupling, and co-citation analyses. *Scientometrics.* 2015;105:2071-2087.

**9.** Nath S, Thomson WM, Baker SR, Jamieson LM. A bibliometric analysis of Community Dentistry and Oral Epidemiology: Fifty years of publications. *Community dentistry and oral epidemiology.* 2024;52(2):171-180.

**10.** Hjellbrekke J. *Multiple correspondence analysis for the social sciences*: Routledge; 2018.
