# Supplementary material for: Mapping the landscape of rural cancer research: a global bibliometric analysis
Source: Cancer Causes Control. 2025 Dec 27;37(1):5. doi: 10.1007/s10552-025-02086-0 (PMC12743660; doi:10.1007/s10552-025-02086-0)
Supplement: Supplementary file 2 — Supplementary file2 (DOCX 16 KB) [file 10552_2025_2086_MOESM2_ESM.docx]

**Supplementary Information 2 (S2)**

**Overview of data completeness and included documents in the final dataset**

| **Description** | **Total (%)**  **(n = 15722)** |
| --- | --- |
| **Data completeness** |  |
| Authors | 15,722 (100.0) |
| Document Type | 15,722 (100.0) |
| Journal | 15,722 (100.0) |
| Language | 15,722 (100.0) |
| Publication Year | 15,722 (100.0) |
| Title | 15,722 (100.0) |
| Total citation | 15,022 (100.0) |
| Overall affiliation ^a^ | 15,622 (99.36) |
| Authors’ institution | 14,421 (91.72) |
| Authors’ country | 15,501 (98.59) |
| Corresponding author country | 14,484 (92.13) |
| Abstract | 14,929 (99.52) |
| Author keywords | 11,885 (75.59) |
| DOI | 14,887 (94.69) |
| **Languages** |  |
| English | 15005 (95.4) |
| Chinese | 331 (2.11) |
| Spanish | 118 (0.75) |
| German | 65 (0.41) |
| French | 62 (0.39) |
| Russian | 57 (0.36) |
| Portuguese | 38 (0.24) |
| Polish | 11 (0.07) |
| Japanese | 7 (0.04) |
| Turkish | 7 (0.04) |
| Korean | 6 (0.038) |
| Italian | 4 (0.025) |
| Croatian | 2 (0.013) |
| Slovenia | 2 (0.013) |
| Romanian | 2 (0.013) |
| Hungarian | 1 (0.006) |
| Malay | 1 (0.006) |
| Persian | 1 (0.006) |
| Serbian | 1 (0.006) |
| Ukrainian | 1 (0.006) |

*^a^ Only counted as missing if all details (i.e. country, institution) are missing or if information could not be extracted.*
